# Determinants of Undernutrition among Young Children Living in Soth Nikum District, Siem Reap, Cambodia

**DOI:** 10.3390/nu11030685

**Published:** 2019-03-22

**Authors:** Sonia Blaney, Lylia Menasria, Barbara Main, Chhea Chhorvann, Lenin Vong, Lucie Chiasson, Vannary Hun, David Raminashvili

**Affiliations:** 1 École des sciences des aliments, de nutrition et d’étude familiale, Université de Moncton, 18 avenue Antonine-Maillet, Moncton, NB E1A 3E9, Canada; lyliamenas@gmail.com; 2 Public Health Specialist, Guelph, ON N1E 6Y8, Canada; mainbarbara@gmail.com; 3 National Institute of Public Health, Phnom Penh 12203, Cambodia; cchhorvann@niph.org.kh; 4 Independent consultant, Phnom Penh 12203, Cambodia; leninvong17@gmail.com; 5 Direction du mieux-être, Ministère du développement social, 1780 rue Water, Miramichi, NB E1N 1B6, Canada; lucie.chiasson@gnb.ca; 6 World Vision Cambodia, #20 Street 71 Tonle Bassac, Chamkar Morn, Phnom Penh 12203, Cambodia; Vannary_Hun@wvi.org (V.H.); David_Raminashvili@wvi.org (D.R.)

**Keywords:** nutritional status, young children, determinant, dietary intake, Cambodia

## Abstract

Background: Child undernutrition is of public concern in Cambodia. An understanding of factors influencing child nutritional status is essential to design programs that will reduce undernutrition. Using the UNICEF conceptual framework of causes of malnutrition, our research investigates the relationship between nutritional status of children aged 6–23 months and its immediate and underlying determinants. Methods: Baseline data from a cluster-randomized controlled trial aiming to assess the impact of the promotion of optimal feeding practices combined or not with the provision of local foods among 360 children 6–23 months of age were used. Anthropometry and biochemical measurements were performed at baseline. Data on each determinant of undernutrition were collected through interviews and direct observations. Results: Our results show that the degree of satisfaction of proteins and zinc requirements as well as the access to improved water sources and sanitation were positively associated with length-for-age, while having a better health status and a higher degree of satisfaction of energy, protein, zinc, and iron requirements were associated to an improved weight-for-length. Only child health status was associated to ferritin. Conclusion: Our results reiterate the importance of improving child diet and health status, but also the access to a healthy environment to ensure an optimal nutritional status.

## 1. Introduction

Worldwide, childhood malnutrition remains of concern, including micronutrient deficiencies. The global nutrition report indicates that 24% of children under-five are stunted and 8% wasted [1]. According to the World Health Organization, iron deficiency is the most common and widespread nutrition disorder. Children and women in developing countries are particularly affected [2].

The UNICEF conceptual framework of malnutrition illustrates the multisectoral nature of nutrition problems [3]. According to this framework, immediate determinants of nutritional status are inadequate dietary intake and diseases that are determined by underlying factors, namely access to food, care, quality health services and healthy environment in turn, influenced by basic causes acting at the societal level.

Over the past decades, attempts have been made to raise awareness about the multisectoral aspect of malnutrition and of the importance of involving sectors other than health. In fact, as demonstrated by the UNICEF framework, malnutrition is determined by a range of factors relying on the implementation of interventions by a multitude of sectors [3]. This multisectoral approach has resurged recently, given the rising of more favorable conditions for initiating projects [4,5]. Likewise, country assessments of nutrition problems are increasingly involving more and different sectors and a growing body of evidence is available about what works or not for improving nutritional status of populations. At the country level, governments and development partners are looking to determine where the problem lie and its causes to develop appropriate multisectoral strategies and programs to ensure optimal impact on the nutritional status of vulnerable populations [5].

To our knowledge, the relationship between young child nutritional status and its multiple determinants in that same study has rarely been assessed. In Gabon, using the UNICEF’s conceptual framework of malnutrition, immediate and underling determinants of young child nutrition status have been investigated [6]. Results have shown that feeding practices and access to improved water and sanitation were the best predictors of length-for-age Z-score (LAZ) explaining 14% of its variance. Recently, in Bangladesh and Vietnam, as part of a development program evaluation, the association between young children’s nutritional status and some of its determinants has been assessed [7]. The results have shown that household food security or having attended at least four prenatal visits, better caregiver nutrition knowledge and hygiene practices were positively associated to the height-for-age (HAZ) among children under five in both countries.

In Cambodia, as well as in other Asian countries, malnutrition is of concern. Stunting and wasting respectively affect 32% and 10% of Cambodian children 0–59 months old [8]. Moreover 55% are anemic whereas the prevalence of inadequate zinc intake ranged between from 15% to 25% [8,9]. An analysis of the 2005 Cambodia Demographic and Health Survey (DHS) has shown that the consumption of animal-source foods was associated to a reduction of the risk of being stunted and underweight among children 12–59 months old [10]. Results of our research conducted in Soth Nikum district of Siem Reap province have also shown that the quality of the 6–23 months old child diet is limited, while only a few appeared to be healthy. In particular, data have revealed that energy, iron, and zinc requirements were not fulfilled, while the degree of satisfaction of protein requirements exceeds almost double the needs [11]. These findings are in accordance with others, which revealed inadequate nutrient intakes (especially iron and zinc) among Cambodian children [12,13,14]. Yet, although these studies shed some light on the situation on immediate determinants of young child nutritional status, it is necessary to extend the investigation to understand the causes of poor dietary intake and health status, as well as the relationship between immediate and underlying determinants and child nutritional status [3]. Such analysis will help shape future investments to improve the nutrition situation among young children.

Therefore, to get a better understanding on which determinants are mostly associated with child undernutrition in Cambodia, this research aims to investigate immediate and underlying determinants of young child nutritional status among children 6–23 months old living in the Soth Nikum, Siem Reap. The UNICEF conceptual framework of malnutrition is used for this purpose [3]. This paper complements our previous publication which has shown that nutritional status, dietary intake and health status of young children living in Soth Nikum district were not optimal [11].

## 2. Materials and Methods

### 2.1. Study Area

Cambodia is located in Southwest Asia in a tropical zone with a hot temperature throughout the year. In 2015, the population was estimated at 15.7 million with around 80% living in rural areas [15]. The literacy rate was 83% for men and 66% for women in 2009. In 2015, the gross national product per capita expressed in power purchasing parity was approximately USD 3500. Agriculture, largely rice production, is the country’s main economic activity.

The study was conducted in Soth Nikum operational district of Siem Reap province within the program area of an international non-governmental organization (NGO), which was looking to initiate a new nutrition program. The latest provincial data indicate 35.9% of children under-five years of age are stunted, 9.5% wasted, and 55.5% anaemic [8]. The NGO’s operational area covers three administrative districts, namely Soth Nikum, Svay Leu, and Chikreng.

### 2.2. Study Design and Sample

The data used for this analysis are from the baseline survey from a cluster-randomized controlled community trial. More information on the research methodology is available in Menasria et al. [11]. In short, all villages located in the coverage area were listed and 14 out of a total of 325 were randomly selected. In each village, all households with a child between 6 to 23 months of age were invited to participate. Exclusion criteria were children with (1) severe anemia or malnutrition, and (2) congenital malformations. If these symptoms were detected, the child was excluded and referred for treatment to the nearest health facility. The sample size was defined to detect a mean improvement of 1 g/dL of hemoglobin level among each of the three groups of children (control group, intervention groups 1 and 2) between baseline and at 6 months of study (endline) implementation assuming 90% power and a 5% significance level. The minimum sample size was 80 children in each of the three arms [16], but we aimed for 120 children/arm because attrition was anticipated during the farming season (May to October), which overlapped with our trial.

### 2.3. Data Collection

Enumerators were recruited locally based on the following criteria: reading and writing abilities, familiarity with the study area, proficiency in the local language, and experience with nutrition surveys. All enumerators received a three-day training on survey methodology and tools. For enumerators assigned to collect anthropometry data, a standardization exercise was performed to ensure quality of measurements. All survey tools were translated into the local language (Khmer) and pretested in communities near the training center before data collection commenced.

#### 2.3.1. Anthropometry and Biochemical Data

All children and each mother/caregiver were weighed with minimal clothing using an electronic scale (UNISCALE, 150 ± 0.1 kg). For children, length was taken in a lying position with a standardized board (UNICEF, 130 ± 0.1 cm) while adult’s height was measured standing (Quick Medical, Issaquah, WA, USA, 230 ± 0.1 cm).

A trained health team from the National Institute of Public Health collected blood from children in two tubes (3–4 mL per tube). The first tube contained an anticoagulant (ethylenediaminetetraacetic acid or EDTA) for hemoglobin testing. The second one was for ferritin and C-reactive protein testing. All specimens were kept in Cool boxes and sent to the National Institute of Public Health laboratory in Phnom Penh for analysis.

#### 2.3.2. Dietary Intake

Information was collected on child dietary intake through three quantified 24-h recalls carried out during two week days and one weekend day. Standardized local utensils, bowls, and cups were used to assess the child food quantities. Information on vitamin and mineral intake as well as food supplementation was collected. The number of times the child was breastfed over the 24 h period was recorded.

#### 2.3.3. Health Status

The presence of signs of illnesses (diarrhea, fever and symptoms of acute respiratory infections) during the past two weeks was recorded. A stool sample was collected from each child. Containers with a preservative and the child’s code were distributed to the caregivers with instructions on how to collect and store the sample. The filled containers were then collected from the caregiver and transported to the laboratory.

#### 2.3.4. Health and Feeding Practices

Data on health practices, namely child immunization coverage, provision of deworming and vitamin A capsule in the past six months, as well as on the use of a mosquito net the previous night were gathered. This questionnaire was based on the 2104 Cambodia DHS standard questionnaire [8]. Information on the age of introduction of foods as well as on breastfeeding practices, the number of meals and food diversity (including fortified foods) in the past 24 h by the child was also collected.

#### 2.3.5. Household Food Security

Household food security status was assessed using the Household Food Insecurity Access Scale (HFIAS), which has been pretested and adapted for use in Cambodia [17,18].

#### 2.3.6. Socio-Demographics and Access to Healthy Environment

Socio-demographic data were collected with a questionnaire through an interview with the head of household and the child’s mother/caregiver on the followings: Age, sex, and education level of household members, asset ownership, housing characteristics, household size, child’s birth weight, and access to an improved water source and sanitation.

A summary of all data collected and methods used for their respective collection is presented in Table 1.

### 2.4. Data Analysis

#### 2.4.1. Nutritional Status

For children under-five years of age, length-for-age (LAZ), and weight-for-length (WLZ) Z-scores were derived with the WHO Anthro 2011 software (version 3.2.2, Geneva, Switzerland). Children with indices below −2 Z-score from the median values of the standards were considered undernourished. In caregivers/mothers, a body mass index (BMI) below 18.5 and over/equal to 25 indicated underweight and overweight respectively [19]. Child-birth weight was assigned in one of two categories namely (1) a birth weight below 2.5 kg was considered inadequate and; and (2) a birth weight at or above 2.5 kg was considered adequate.

Hemoglobin was tested using the fluorescence flow cytometry technology (Sysmex machine, model XT-1800i) while ferritin was analyzed using Cobas e601 5 Analyzer (Roche Diagnostics, Risch-Rotkreuz, Switzerland). A hemoglobin level below 11 g/dL indicates anemia among children, while a value below 12 ug/L highlights limited/no iron storage for ferritin [20]. The C-reactive protein (CRP) was also tested using the immunoturbometric method (Bayer machine, model 7180) to assess the presence of inflammation. Children with a CRP value above 5 mg/L were excluded, given the potential impact on ferritin measurement [20].

#### 2.4.2. Dietary Intake

Data on child dietary intake were analyzed using Nutrific (Version 1.1, Université Laval, Québec, QC, Canada) to calculate the daily energy and nutrient intakes, namely protein, iron, zinc, and vitamin C. The nutritive value of foods not originally included in the software were added to the database from information on labels or from other sources such as the Association of South East Asian Nations table and the Sustainable Micronutrient Interventions to Control Deficiencies and Improve Nutritional Status and General Health in Asia (Smiling project) Food composition table for Cambodia [21,22].

Based on its daily breastfeeding frequency [23,24], each breastfed child was classified into one of the following categories: low: 1–4 times/day, average: 4–6, and high: 7 or more. The daily breastmilk intake was then estimated based on Dewey and Brown’s classification regarding low, average and high intake of breastmilk [25]. Mean energy, protein, iron, zinc, and vitamin C intake from other foods and beverages (excluding breastmilk) consumed over the three-day period were also estimated for each child and added to record total energy and nutrient intakes. Total intakes were thereafter compared to the estimated daily energy and nutrient requirements using international recommendations [26,27,28]. Iron requirements were estimated based on a diet with bi-availability of iron at 10% and bioavailability of zinc at 12% was used [27].

#### 2.4.3. Health Status

Health status of each child was assessed based on whether or not he presented with signs of diarrhea, fever or an acute respiratory infection (ARI) during the 14 days preceding the study. A score was assigned to each child as follows: Zero (0) if child was not sick; one (1) if he presented signs of either diarrhea or fever or ARI; two (2) if he had signs of two out of the three illnesses; and three (3) if he had signs of all of them.

The child was also assigned a dichotomic score based on whether (score of one (1)) or not (score of zero (0)) he had intestinal parasite at the time of the survey. The presence of intestinal parasites was examined microscopically using the formalin-ether sedimentation technique [29].

#### 2.4.4. Household Food Security

Based on the respondent’s reply to each of the nine questions of the HFIAS, a score between 0 and 27 was assigned to each household to assess its access to food.

#### 2.4.5. Feeding and Health Practices

Child feeding practices were assessed using WHO core indicators for children 6–23 namely currently breastfed or not, minimum dietary diversity and minimum meal frequency and minimum acceptable diet [30,31]. A score of zero (0) or one (1) was assigned to each child based on whether or not he met the WHO recommendations for the three aforementioned indicators. Moreover, current breastfeeding status of the child was also determined and categorized as if he was breastfed (score of 1) or not (0). To assess health practices, a score was developed using data on immunization. If the child was immunized according to recommendations at the age of one (1) year, a score of one (1) was attributed, while a score of zero (0) was given if not.

#### 2.4.6. Access to a Healthy Environment

A score of zero (0), one (1), or two (2) was assigned to each child depending on whether his household had access or not to both, an improved sanitation or water source, respectively. International definitions were used to categorize each household access to these facilities [32].

#### 2.4.7. Socioeconomics

A factor analysis using the principal axis factoring was performed to define a socioeconomic score for each household. Initially, 18 items on ownership of assets and housing conditions were used to develop the score. On final, the score considered 11 items namely ownership of a fan, television, wardrobe, motorcycle and bicycle, watch, boat, mobile phone, radio, having electricity, and number of rooms in the house. All together, they explain 29.9% of the total variance and the composition of the first factor. The Kaiser-Meyer-Olkin Test was used to assess the suitability of the data for the factor analysis. The result of the test was 0.803, which is satisfactory [33].

### 2.5. Statistical Analysis

Anthropometric Z-scores as well as data on food and nutrient intakes were analyzed with SPSS (version 21, Statistical Package for the Social Sciences, Statistics for Windows, Version 21.0, IBM Corporation, Armonk, NY, USA). Normality in the distribution pattern was examined by visual inspection of the probability plots and with the Kolmogorov-Sminov test. The homogeneity of the variance was assessed with the Levene test. When necessary, logarithmic transformations (such as on data on ferritin and on the degree of satisfaction of nutrient requirements) were applied to obtain normal distribution patterns and homoscedasticity. For continuously distributed variables, ANOVA and post hoc Student–Newman–Keuls or Bartlett’s tests were used. Chi-square tests were performed to assess differences between proportions. Pearson and Spearman correlations were conducted to assess association between continued and ordinal data. Linear regressions using a stepwise approach were used to assess the relationship between child nutritional status and potential determinants. For linear regressions, the threshold was considered significant when the *p*-value was below 0.10, while for other tests, a *p* value below 0.05 was used. Correlation analyzes between indicators of nutritional status were conducted with MPlus software (8th version, Computer Software, Los Angeles, CA, USA, Muthén and Muthén, 1998–2017).

### 2.6. Ethical Approval

The study was approved by the National Ethics Committee for Health Research of the Ministry of Health (#367NECHR), Kingdom of Cambodia and the Ethic Committee on Human Subjects Research of the Université de Moncton, New Brunswick, Canada (#CER161125).

## 3. Results

Overall, with the exception of the proportion of children born with low birth weight, there was no significant difference among sociodemographic characteristics among all groups (Table 2).

In our population, the prevalence of stunting and wasting was 19.1% and 8.4% while 76.1% and 37.5% of children had low levels of hemoglobin and ferritin, respectively.

Mean LAZ and WLZ were lower among the children aged 18–23 months old but the hemoglobin value was significantly higher in this group as compared to children 6–11 months and 12–17 months old (Table 3). Moreover, the mean LAZ was lower among children born with a low birth weight, while the mean WLZ was lesser among those whose mothers had a BMI below 18.5. Children aged from 12 to 17 months, male, and those born with a low birth weight had the lowest mean ferritin values.

Results of bivariate analyses indicate no association between anthropometric Z-scores and indicators on iron status (Table 4). LAZ was positively correlated to the degree of satisfaction of energy requirements (DSER) but negatively associated with the degree of satisfaction of protein requirements (DSPR) and the presence of intestinal parasites. There was a negative association between WLZ and DSPR, as well as with the health status, as measured by the presence of signs of three illnesses in the past two weeks. Hemoglobin level was positively correlated with the ferritin value, DSER, and DSPR, but negatively correlated with breastfeeding.

The DSER was positively correlated to the DSPR, the degree of satisfaction of iron (DSIR) and of zinc requirements (DSZR) as well as with breastfeeding status (Table 4). Receiving the minimum acceptable diet was positively correlated with breastfeeding status, the DSPR, DSIR, DSZR, and the degree of satisfaction of vitamin C requirements (DSCR), but negatively with household food security. Access to a healthy environment was not associated with the child’s health status, but it was negatively correlated with access to foods.

Results of the linear regression models indicate that predictors of LAZ were the DSPR and the DSZR as well as being breastfed and having access to improved water source and sanitation (Table 5). The DSPR and being breastfed were negatively associated with LAZ while the DSZR and having access to a healthy environment were positively associated with LAZ. Being a male and older were both negatively associated with LAZ. These determinants together with child characteristics explained 17.0% of the variance associated with LAZ.

With the exception of DSCR, the degree of satisfaction of energy and nutrient requirements as well as the health status were all predictors of WLZ but the DSPR and health status was negatively associated while there was a positive association between DSER, DSIR, DSZR, and WLZ (Table 5). All determinants explained 15.5% of the variance associated with WLZ. The DSZR explained around 6% of the variance associated with LAZ and WLZ.

Health status (having presented signs of different illnesses in the past two weeks) was the only predictor of ferritin. Together with being a male and born with a low birth weight, it explained 4.1% of its variance. There was no determinant associated with the hemoglobin level with the exception of being aged 18–23 months (Table 5).

## 4. Discussion

Undernutrition among young children remains of public concern in low and middle-income countries such as Cambodia. An understanding of determinants of this situation is essential to make successful investments to ensure optimal child growth and development.

Results of this research show that predictors of the length-for-age z-score in young children are the degree of satisfaction of proteins and zinc requirements, the access to a healthy environment and the breastfeeding status. Health status, the degree of satisfaction of energy, protein, zinc, and iron requirement were the predictors of weight-for-length index. While no firm conclusion can be drawn on the cause-effect relationship between young child nutritional status and its determinants, increasing the degree of satisfaction of energy, iron and zinc requirements and reducing illnesses may help improve WLZ among young children. This result was expected since WLZ reflects short-term conditions. In this context, data on food intake and presence of signs of illnesses were collected at the same time that the WLZ measurement was performed. The presence of illnesses was associated with a reduced WLZ. This can be explained by the reduced appetite caused by the illness, which can affect the child’s weight.

A higher degree of satisfaction of zinc requirements and an improved access to a healthy environment were both positively associated to LAZ. Zinc plays an essential role in many biological processes such as cell growth, and a deficiency in this micronutrient may restrict childhood growth and decrease resistance to infections, which contribute significantly to morbidity and mortality in young children [34,35,36]. It is thus likely that a better satisfaction of zinc requirements would contribute to optimal linear growth and weight gain, and thus, to improved LAZ and WLZ.

With regard to the positive impact of access to healthy environment on LAZ, in spite of the fact that 60% and 80% of households living in rural areas have access to improved water source during the dry and rainy seasons, respectively, still, the access to improved sanitation appears to be limited in this setting. In fact, around 50% of households have no access to improved sanitation facility in rural area [8]. Therefore, in our study area, even though young children may have access to improved water sources, they may not have access to improved sanitation. However, young children are not necessarily using household theses facilities butthey can be used for child’s stools disposal thus reducing risks of fecal-oral diseases. Our results are in line with a recent Cochrane review which has identified a statistically significant effect of WASH interventions on HAZ [37]. In Gabon, Blaney et al. have shown that access to improved water and sanitation explained 10% of the variance associated with LAZ among children 0–23 months [6]. The impact of access to improved water sources and sanitation on child nutritional status may be through the reduction of the risk of diarrhea [38,39], environmental enteropathy [40], and parasite infections [41].

Our results showed that the DSPR was negatively associated with LAZ and WLZ. As observed in our study, as well as by Reinbott et al. [42], children were mainly eating rice, which is a source of low quality protein. In fact, as pointed earlier, the DSPR appeared to be above the needs while it was not the case for energy and other nutrients [11]. This situation has been reported by Millward [43] who highlights that most diets and especially cereal-based diets provide more than adequate amounts of protein requirements. In addition, as far as protein quality is concerned, it is unclear on how well linear growth can occur in the best circumstances for cereal-based diets with minimal animal source foods given the potential dietary limitations of micronutrients as well as the suboptimal protein quality. Millward [43] also underlines that deficiencies in some nutrients such as protein, but also of zinc could inhibit growth. As such, there is evidence that protein deficiencies can occur in the diet, especially for populations consuming diets based on starchy roots or cereals with little or no animal-source foods. For instance, in India, Swaminatha et al. [44] have shown that the largely cereal-based diets among populations, which exhibit high prevalence rates of stunting, appear adequate in terms of protein intakes.

In the Soth Nikum area, a limited diet diversity was most likely a limiting factor of the child nutritional status. An analysis of DHS data from 14 countries including Cambodia, has shown that a higher dietary diversity was strongly associated with a higher HAZ score [45]. Yet, we did not find any correlation between the number of food groups consumed by the child and LAZ and WLZ. However, with the exception of vitamin C, the degree of satisfaction of nutrient requirements were all strongly (*p* < 0.01) and positively associated with the number of food groups consumed in the past 24 h (results not shown).

Being breastfed at the time of the study was negatively associated with LAZ. It is possible that there was an overreliance on breastfeeding among our population, which may have competed with the provision of complementary foods. It is also possible that there was a delay in the introduction of complementary foods among children, which may have led to difficulties to introduce them. In fact, 10.0% of children 6–23 months old part of our study were not yet given soft, semi-solid, or solid foods, while only 15.2% of these had 1–2 meals the previous day. Similar observations were reported from an analysis of data from 19 countries by Caulfield et al. [46]. Data from nine of the 19 indicate that older still breastfed children were lighter and/or shorter than no longer breastfed children. This situation deserves further research to ensure that quality complementary foods are provided timely and in sufficient quantity along with the continuation of breastfeeding.

In accordance with the findings of this study, results of previous research in Cambodia have shown that the prevalence of stunting increased with age and higher among male children [8,45].

The positive association between ferritin and a poor health status may be explained by high ferritin level in presence of acute or chronic infection. It has been reported that the synthesis of ferritin is stimulated by infection. To control partially for that situation, it was suggested that, in addition to ferritin, an independent indicator of the acute phase response, such as CRP to be measured [20,47]. CRP was thus assessed in this study and children with elevated levels were excluded (5% of children) from the data analysis. However, we did not control for chronic or sub-clinical infection. As such, we could have added AGP (alpha1-acid-glycoprotein) as it indicates chronic infection and it may better reflect the changes in the concentration of ferritin during infections [48].

An association between low level of ferritin and being born with a low birth weight has been reported previously [49,50]. Authors explained this finding by the fact that ferritin stores of low birth weight children may have been depleted by rapid growth in early infancy, which may have increased the risk of iron deficiency. With regards to hemoglobin, the absence of relationships between the determinants under study may be explained by the fact that levels may also be influenced by other factors such as vitamin B12 intake, genetic abnormalities such as thalassemia and other hemoglobinopathies and by infections. In the Soth Nikum operational district, as mentioned previously, CRP was assessed to check for infections among children and those with elevated CRP were excluded from the data analysis. Given the small proportion of children that were excluded (5%), their removal has not likely induced any bias. Moreover, feces were collected to assess the presence of intestinal parasites and in our regression models, this variable was considered. However, we did not assess the presence of hemoglobinopathies, which may lead to lower hemoglobin level in our group. Nevertheless, available data suggest that hemoglobin E (HbE) and β-thalassemia, is also likely and even common among Cambodian children [8,51]. Even though many (32%) have normal hemoglobin, around 25% of them are affected by either heterozygote (24%) or homozygote HbE (3%). An additional 23% have other forms of hemoglobinopathy [8].

The absence of a relationship between the access to the minimum diet and young child nutritional status may be due to the fact that the current situation may not reflect past practices. Food practices among young children evolve with their age and change significantly in the first two years. As expected, benefiting from the minimum acceptable diet was positively associated with the satisfaction of energy and nutrient requirements. Lastly, the lack of association between household food security and child nutritional status has also been reported elsewhere [6,52,53]. This may be attributed to the fact that the measurement is at the household level and may not reflect access to food for young children who may be privileged by the family with regard to intra-household food distribution, or even benefit from special foods.

In addition to the aforementioned limitations, although significant efforts were dedicated to assess food and nutrient intakes, and in spite of the fact that 24 h recall were included to minimize memory recall bias, estimating young child dietary intake remains a challenge when small amounts of food are consumed. Unlike WLZ, LAZ is the result of past insults related to food intake and illnesses. Therefore, the current situation may not reflect past practices, which may also be a limitation of this analysis, which was cross-sectional and did not capture past practices and events.

## 5. Conclusions

Child malnutrition is a public health problem in rural Siem Reap, Cambodia. Our study shows that determinants of undernutrition included dietary intake, health status, access to an improved water source and sanitation and breastfeeding status. Investments in these sectors could contribute to improve young child nutritional status. For future research, we suggest to collect more information on consumption patterns over time, the presence of hemoglobinopathies, infections, and on vitamin A intake so as to get a better understanding of causes of poor nutritional status.

## Figures and Tables

**Table 1:** Summary of the data collected and methods used.

Type of Data	Methods for Data Collection
Anthropometry	Direct measurement
Hemoglobin and ferritin	Venous blood collection
Dietary intake	Interview for 24-h recalls (3)
Health Status	Interview to collect data on signs of diarrhea, fever or acute respiratory infection in the 14 days preceding the study, and C-reactive protein
Health and feeding practices	Interview to collect data on breastfeeding status of the child, minimum dietary diversity and meal frequency the last 24-h using a standardized questionnaire
Household food security	Interview using the household food insecurity access scale
Access to healthy environment	Interview using a standardized questionnaire to collect data on household access to improved sanitation and water source
Socio-demographics	Interview using a standardized questionnaire to collect data on household ownership of assets and housing conditions.

**Table 2:** Description of the population.

Characteristics	*n*	%	Mean (±SD)
**Child’s characteristics**			
Age group (months)	346		NA
6–11		39.3	
12–17		36.1	
18–23		24.6	
Gender	346		NA
Female		50.9	
Male		49.1	
**Caregiver’s characteristics**			
Age	346		30.1 ± 9.6
Education level (years) *	345		
None		27.5	
1–6		50.7	
≥7		21.7	

* Data were missing for one child.

**Table 3:** Mean (± standard deviation/SD) for anthropometric and iron status indicators * by child, caregiver and household characteristic.

	Anthropometric Indicators **			Iron Status Indicators			
	*n*	LAZ	WLZ	*n*	Hb	*n*	Ferritin
	**Child characteristics**						
Age groups (months)							
6–11	135	−0.67 ^a^	−0.60 ^a^	109	9.75 ^a^	112	27.61 ^a^
		(1.43)	(1.06)		(2.2)		(27.32)
12–17	125	−1.25 ^b^	−0.76 ^a^	112	9.77 ^a^	114	17.29 ^b^
		(0.92)	(0.93)		(1.24)		(15.9)
18–23	85	−1.47 ^b^	−1.04 ^b^	75	10.53 ^b^	76	22.87 ^a^
		(1.17)	(0.93)		(1.15)		(19.4)
Gender							
Female	176	−0.96	−0.76	155	10.11	159	23.53 ^a^
		(1.33)	(0.9)		(1.96)		(20.48)
Male	169	1.19	−0.78	141	9.78	143	21.39 ^b^
		(1.14)	(1.09)		(1.23)		(23.59)
Birth weight (kg)							
<2.5	31	−1.61 ^a^	−1.10	27	9.69	27	15.36 ^a^
		(0.94)	(0.78)		(1.33)		(13.14)
≥2.5	304	−1.05 ^b^	−0.73	260	9.98	266	23.37 ^b^
		(1.17)	(1.02)		(1.72)		(22.86)
	**Caregiver characteristics**						
Education level (years)							
None	95	−1.15	−0.70	80	9.98	82	20.87
		(1.12)	(0.92)		(1.25)		(16.97)
1–6	174	−1.03	−0.78	151	9.84	152	23.57
		(1.19)	(0.98)		(2.02)		(24.53)
≥7	75	−1.09	−0.84	64	10.17	67	22.39
		(1.5)	(1.14)		(1.13)		(21.63)
Body-mass index							
<18.5	29	−1.30	−1.15 ^a^	27	9.89	27	19.41
		(0.99)	(1.04)		(1.22)		(14.98)
≥18.5	235	−1.10	−0.74 ^b^	202	9.99	202	22.71
		(1.18)	(0.98)		(1.19)		(21.35)
	**Household characteristics**						
Household size (persons)							
1–3	84	−1.14	−0.75	77	9.56	78	19.85
		(1.01)	(1.04)		(2.54)		(16.25)
4–6	196	−1.05	−0.80	162	10.09	167	23.65
		(1.33)	(1.03)		(1.18)		(24.88)
≥7	65	−1.07	−0.72	57	10.11	57	22.88
		(1.26)	(0.82)		(1.28)		(19.75)
Socioeconomic quintile							
		−1.20	−0.64		9.84		22.58
Lowest	67	(0.96)	(1.09)	58	(1.18)	59	(25.57)
		−1.14	−0.73		9.62		19.83
Second	67	(1.09)	(0.9)	57	(2.89)	57	(16.21)
		−1.09	−0.87		9.94		19.95
Middle	67	(0.96)	(0.85)	55	(1.3)	56	(17.26)
		−0.95	−0.91		10.15		24.85
Fourth	66	(1.19)	(0.98)	61	(1.19)	62	(19.63)
		−1.00	−0.70		10.18		24.87
Highest	67	(1.44)	(1.16)	55	(1.17)	58	(29.41)

* Different letter in the same column indicate significant differences (*p* < 0.05). ** LAZ: Length-for-age; WLZ: Weight-for-length; Hb: hemoglobin.

**Table 4:** Correlation matrix between indicators on nutritional status and its determinants * (*n* = 336).

	LAZ	WLZ	HB	FER	DSER	DSPR	DSIR	DSZR	DSCR	HS1	HS2	FS	IMM	MAD	BF	AHE
LAZ	1															
WLZ	0.194 ^†^	1														
HB	−0.130	−0.023	1													
FER	−0.034	−0.068	0.381 ^†^	1												
DSER	0.112 ^†^	0.072	0.074	0.106	1											
DSPR	−0.174 ^†^	−0.206 ^†^	0.168 ^†^	0.032	0.483 ^†^	1										
DSIR	−0.099	−0.056	0.164 ^†^	0.014	0.147 ^†^	0.702 ^†^	1									
DSZR	−0.008	−0.030	0.100	−0.023	0.596 ^†^	0.827 ^†^	0.724 ^†^	1								
DSCR	0.047	0.027	−0.030	0.096	0.011	0.267 ^†^	0.266 ^†^	0.368 ^†^	1							
HS1	−0.018	−0.195 ^†^	−0.067	0.117	−0.013	−0.020	−0.027	−0.028	0.082	1						
HS2	−0.242 ^†^	0.017	0.065	0.011	−0.109	−0.050	0.002	−0.063	−0.105	−0.045	1					
FS	0.011	0.041	0.016	0.013	0.011	−0.076	−0.097	−0.071	−0.089	0.156 ^†^	0.053	1				
IMM	−0.066	−0.107	0.000	−0.023	−0.035	0.185 ^†^	0.164	0.110	0.023	−0.032	−0.142	0.072	1			
MAD	0.012	−0.067	−0.040	−0.117	0.103	0.174 ^†^	0.208 ^†^	0.241 ^†^	0.297 ^†^	0.011	−0.076	−0.210 ^†^	0.060	1		
BF	0.138	0.081	−0.213 ^†^	−0.025	0.443 ^†^	−0.189 ^†^	−0.455 ^†^	−0.063	0.551 ^†^	0.004	−0.185	−0.039	−0.142	0.325 ^†^	1	
AHE	0.092	0.008	0.017	−0.039	−0.044	0.143 ^†^	0.104	0.053	−0.012	−0.098	0.009	−0.203 ^†^	0.190 ^†^	0.094	−0.017	1

^†^*p* < 0.05; * LAZ: Length-for-age *z*-score, WLZ: weight-for-length *z*-score, HB: hemoglobin, FER: ferritin, DSER: degree of satisfaction of energy requirements, DSPR: degree of satisfaction of proteins requirements, DSIR: degree of satisfaction of iron requirements, DSZR: degree of satisfaction of zinc requirements, DSCR: degree of satisfaction of vitamin C requirements, HS1: score on health status based on the presence of signs of illnesses in the past two weeks, HS2: presence of parasites (yes/no), FS: score on household food security, IMM: score on immunization coverage at 1 year, MAD: score on minimum acceptable diet, AHE: score on access to a healthy environment (improved water and sanitation).

**Table 5:** Regression analyses of nutritional status (LAZ, WLZ, hemoglobin and ferritin) on its determinants (*n* = 296) *: final models.

	LAZ			WLZ			Hb			Ferritin		
	R Change	Beta	*p*	R Change	Beta	*p*	R Change	Beta	*p*	R Change	Beta	*p*
**Constant**	-	2.622	0.001	-	2.686	0.001	-	97.269	0.000	-	1.193	0.000
**DSER**	-	−0.002	0.983	0.008	0.188	0.014	-	0.066	0.298	-	0.074	0.228
**DSPR**	0.010	−0.622	0.000	0.067	−0.690	0.000	-	0.061	0.319	-	0.001	0.988
**DSZR**	0.070	0.550	0.000	0.061	0.252	0.037	-	0.068	0.264	-	−0.018	0.765
**DSIR**	-	0.105	0.265	0.009	0.180	0.063	-	0.025	0.694	-	0.016	0.792
**DSCR**	-	0.035	0.595	-	−0.054	0.394	-	0.006	0.929	-	0.046	0.453
**HS1**	-	0.013	0.808	0.023	−0.160	0.002	-	−0.052	0.396	0.016	0.125	0.038
**AHE**	0.016	0.129	0.014	-	0.056	0.286	-	0.024	0.690	-	−0.035	0.559
**FS**	-	−0.005	0.926	-	0.077	0.142	-	0.037	0.543	-	0.047	0.439
**BF**	0.015	−0.171	0.007	-	−0.063	0.308	-	−0.051	0.479	-	−0.025	0.675
**MAD**	-	−0.012	0.824	-	−0.022	0.680	-	0.036	0.553	-	−0.068	0.263
**Sex (male)**	0.025	−0.158	0.003	-	−0.063	0.239	-	-	-	0.025	−0.158	0.009
**Aged 18–23 months**	-	-	-	-	-	-	0.046	0.215	0.000	-	-	-
**Age**	0.050	−0.248	0.000	-	-	-	-	-	-	-	-	-
**LBW**	-	-	-	-	-	-	-	-	-	0.011	−0.104	0.084
**R^2^**	0.186	-	-	0.168	-	-	0.046	-	-	0.052	-	-
**R^2^ adj.**	0.170	-	-	0.155	-	-	0.043	-	-	0.041	-	-

* LAZ: Length-for-age *z*-score, WLZ: weight-for-length *z*-score, Hb: hemoglobin, DSER: degree of satisfaction of energy requirements, DSPR: degree of satisfaction of proteins requirements, DSIR: degree of satisfaction of iron requirements, DSZR: degree of satisfaction of zinc requirements, DSCR: degree of satisfaction of vitamin C requirements, HS1: health status based on the presence of signs of illnesses in the past two weeks, FS: household food security, BF: breastfed, MAD: minimum acceptable diet, AH: access to a healthy environment, LBW: born with a low birth weight.

## Abbreviations

UNICEF	United Nations Children’s Funds
LAZ	Length for age Z-score
HAZ	Height for age Z-score
DHS	Demographic and health survey
NGO	Non governmental organization
HFIAS	Household food insecurity and access scale
WHO	World Health Organization
WLZ	Weight for length Z-score
BMI	Body-mass index
CRP	C-reactive protein
ARI	Acute respiratory infection
DESR	Degree of satisfaction of energy requirements
DSPR	Degree of satisfaction of protein requirements
DSIR	Degree of satisfaction of iron requirements
DSZR	Degree of satisfaction of zinc requirements
DSCR	Degree of satisfaction of vitamin C requirements
